# Quantifying the reduction in sexual transmission of HIV-1 among MSM by early initiation of ART: A mathematical model

**DOI:** 10.1371/journal.pone.0236032

**Published:** 2020-07-20

**Authors:** Juan Berenguer, Javier Parrondo, Raphael J. Landovitz

**Affiliations:** 1 Unidad de Enfermedades Infecciosas/VIH, Hospital General Universitario Gregorio Marañón, Madrid, Spain; 2 Instituto de Investigación Sanitaria Gregorio Marañón (IiSGM), Madrid, Spain; 3 Parrondo Health, Coslada, Spain; 4 UCLA Center for Clinical AIDS Research & Education, Los Angeles, CA, United States of America; Fred Hutchinson Cancer Research Center, UNITED STATES

## Abstract

**Background:**

We analyzed the effect of time to initiation of antiretroviral therapy (ART) after diagnosis on the probability of HIV-1 transmission events (HIV-TE) in naïve HIV-1-infected men having sex with men (MSM).

**Setting:**

Mathematical model.

**Methods:**

We used discrete event simulation modeling to estimate the probability of HIV-TE in the first 8 weeks after ART initiation; we varied ART initiation from D0 to D28 after simulated “diagnosis”. The model inputs used sexual behavior parameters from the MSM population of the START trial, and transmission rates per-sex act and HIV-1 RNA from recent meta-analyses. HIV-1 RNA decay curves were modeled from the databases of Single (efavirenz [EFV] v dolutegravir [DTG]), Spring-2 (raltegravir [RAL] v DTG), and Flamingo (darunavir/ritonavir [DRVr] v DTG) trials.

**Results:**

We found that the number of HIV-TE per index patient in the first 8 weeks after ART initiation increased linearly for same-day ART to initiation on day 28. Small but statistically significant advantages of integrase strand transfer inhibitors (INSTI) over EFV and DRVr were found.

**Conclusions:**

Rapid, if not same-day initiation of INSTI-based ART to newly diagnosed HIV-infected MSM has the potential for substantial public health benefits related to decreases in HIV-TE.

## Introduction

Initiation of antiretroviral therapy (ART) regardless of CD4+ count reduces the risk of AIDS and non-AIDS-related morbidity and mortality in patients with HIV-1 infection [1]. Additionally, ART and viral suppression reduces the rates of sexual transmission of HIV-1 to negligible levels [2–4], leading to universal recommendation of provision of ART for all persons living with HIV (PLWH) globally [5].

The benefits of early initiation of ART has led to discussions and investigation of accelerated ART initiation, including starting the same day as an HIV-1 infection diagnosis is confirmed. This approach has been associated with higher rates of retention in care, better virologic outcomes and lower mortality in resource-limited settings [6, 7], and shorter times to virologic suppression in high-resource settings [8]. Concerns surrounding rapid initiation of ART include the prevalence of transmitted resistance because it could influence the choice of ART regimen, and the readiness of patients with newly diagnosed HIV infection to initiate ART rapidly [9]. WHO recommended in 2017 that rapid ART initiation, defined as within 7 days from the day of HIV diagnosis, should be offered to all people living with HIV following a confirmed HIV diagnosis and clinical assessment; including the offer of same-day initiation where there is no clinical contraindication and such resources are available [10]. The offer of rapid initiation, including same-day ART, may further reduce transmission to HIV-negative partners; however, data confirming this benefit is lacking.

Because a clinical trial to analyze the effect of time to initiation of ART after diagnosis on the probability of sexual transmission of HIV-1 would be infeasible, we aimed to evaluate this issue using a mathematical model.

## Materials and methods

We modelled the probability of sexually transmitted HIV-1 infection depending on the the time to ART initiation in HIV-1-infected men having sex with men (MSM).

### Structure of the model

We used a discrete event simulation (DES) model using Microsoft Excel (2016) and Visual Basic for applications (VBA) that has been previously described. The model structure is shown as supplementary material (S1 Fig). With this model, we found that regimens based in the integrase strand transfer inhibitors (INSTIs) dolutegravir (DTG) or raltegravir (RAL), provide advantages over both efavirenz (EFV)- and darunavir/ritonavir (DRVr)-based regimens for initial ART in the reduction of early HIV-1 transmission risk from anal intercourse among MSM.

In brief, input variables in the model included sexual behaviour, HIV-1 RNA levels over time on ART, and transmission risk per sexual act by HIV-1 RNA level. Sexual behavior during the first 8 weeks after initiation of ART, including the number of HIV-negative sexual partners, and the frequency of condomless insertive and receptive anal intercourse per partner was simulated from the MSM population of the INSIGHT Strategic Timing of Antiretroviral Treatment (START) Trial [11]. We assumed no changes in numbers of partners or rates of condomless sex over the 8-week horizon time of observation, something that has been corroborated in a new substudy on sexual behaviour in the START trial [12]. Fractional polynomial regression of repeated measurements of HIV-1 RNA from baseline up to week 24 from the databases of the Single, Spring-2, and Flamingo trials were used to model HIV-1 RNA decay curves for each simulated patient and ART regimen (INSTI, EFV, and DRV/r). The HIV-1 transmission rates per sexual exposure for each HIV-1 RNA level were obtained from the Wilson mathematical model [13], in which the risk of transmission of HIV-1 based on HIV-1 RNA was modelled from the results of the Rakai study of HIV transmission in heterosexual couples [14], in which each ten-fold increment in viral load is associated with a 2.45-fold (95% CI 1.85–3.26) increase in the risk of HIV transmission per sexual contact, according to the equation:

$$\beta_{1}=2.45^{\log_{10}\left(V_{1}/V_{0}\right)}\beta_{0}$$

where β_0_ is the probability of HIV transmission from a person with a baseline viral load V_0_, and β_1_ is the transmission probability corresponding to any other viral load V_1_, whether above or below the baseline. V_0_ (lower and upper uncertainty bounds) is 4.3 x 10^−5^ (1.6 x 10^−5^–11.6 x 10^−5^) and corresponds to the expected transmission probability per male to female sexual act in a serodiscordant partnership, assuming the HIV-infected male has a viral load of 10 copies per milliliter. As the Wilson equation used was derived for serodiscordant heterosexual couples, probabilities were adjusted by using the Odds Ratio of the type of sexual relationship (receptive or insertive anal intercourse) versus a receptive vaginal intercourse that were obtained from a recent systematic review by Patel et al. [15].

We did not consider the use of PrEP or the presence/impact of untreated sexually transmitted infections in the model.

### Design

The horizon of observation was the 8-week period following initial HIV-1 diagnosis. Five million theoretical individuals were modeled to determine the number of secondary sexually transmitted HIV-1 infections arising from MSM initiating 3-agent suppressive ART regimens based on INSTI (DTG or RAL), EFV or DRVr. These 5 million theoretical patients were cloned 87 times; 29 clones for each regimen type initiated therapy at 29 different time points (one clone each from day 0 to day 28) for INSTI-, EFV, and DRV-based therapy. A graph describing the study design is shown as supplementary material (S2 Fig).

### Model outputs

The model outputs provide the following parameters for the simulated patients engaging in condomless sex with an HIV-serodifferent partner (approximately 20% of the total population): 1) The number of simulated HIV-1-negative partners, 2) The number of simulated sexual encounters, and 3) The number of simulated HIV-1 transmission events.

### Sensitivity analyses

Given the uncertainty of the β_0_ parameter in the Wilson equation, two sensitivity analyses were performed using the lower and upper 95% confidence interval (CI) boundaries of this parameter. As retention in care after rapid initiation of ART is a relevant issue, particularly in resource limited settings, we carried out a third sensitivity analyses considering that 10% of patients never presented for care after the first visit assuming that during the follow-up they did not take the medication [16].

## Results

### Simulated sexual activity

The simulated sexual activity over the full 8-week period after initiation of ART in the three arms (INSTI, EFV, and DRV/r) is shown in **Table 1**.

**Table 1:** Simulated sexual activity and HIV-1 transmission events after initiation of ART, for the full week 0 to 8 period, in the three treatment arms (INSTI, EFV, and DRV/r) parametrized according to the sexual risk behavior questionnaire in MSM recruited in the START trial*.

	ART regimen		
Simulated sexual activity	INSTI	EFV	DRV/r
Patients who initiated ART	5,000,000	5,000,000	5,000,000
Patients who engaged in CLSD (20% of those initiating ART)	1,000,000	1,000,000	1,000,000
Partners of patients who engaged in CLSD	9,613,268	9,598,679	9,621,582
Sexual encounters in patients who engaged in CLS-D	29,599,902	29,642,836	29,615,359
Partners per patient who engaged in CLSD	1.92	1.92	1.92
Sexual encounters per partner in patients who engaged in CLSD	3.08	3.09	3.08

*MSM population is based in sexual activity report on START trial (only 20% of the MSM population have condomless sex with an HIV-1-discordant status partner). In addition, a very small number of intercourse events among MSM in the START trial were reported to be with women.

Abbreviations: ART, antiretroviral therapy; INSTI, integrase strand transfer inhibitor; EFV, efavirenz; DRV/r, darunavir/ritonavir; MSM, men who have sex with men; CLSD, condomless sex with an HIV-1-discordant status partner.

Overall, during the 8 weeks after the initiation of ART, per 5 million simulated MSM patients initiating ART, 1 million (20%) engaged in condomless sex; those patients had approximately 9.6 million simulated sexual partners, resulting in approximately 29.6 million simulated condomless sex acts. The number of partners per patient who engaged in condomless sex act with an HIV-negative partner was 1.92; and the number of simulated condomless sex acts with an HIV-negative partner per simulated patient who engaged in condomless sex was 3.08–3.09.

### Simulated HIV-1 transmission events

**Fig 1:**
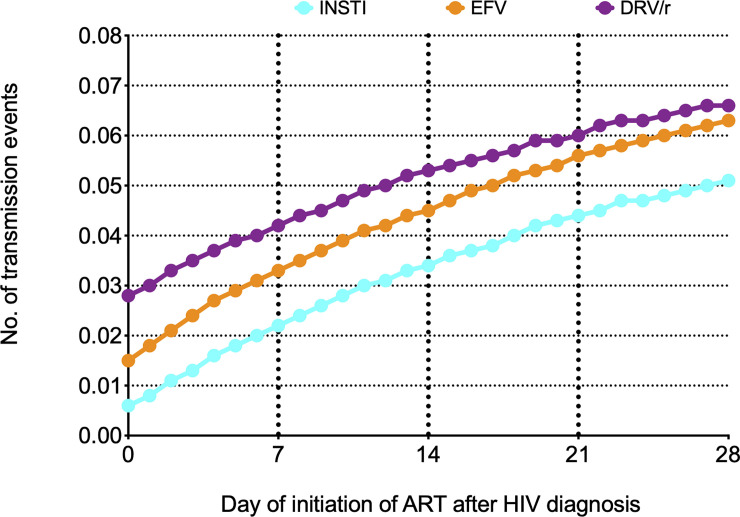
Number of incident simulated HIV-1 transmission events per patient during the full 8-week period after initiation of ART by day of initiation of ART.

The number of incident simulated HIV-1 transmission events per patient during the full 8-week period after initiation of ART by day of initiation of ART are shown in Fig 1, and supplementary material (S1 Table).

In brief, for patients initiating ART on day 0 or day 28, the number of transmitted infections per patient varied between 0.012 and 0.098 for patients initiating INSTI, between 0.030 and 0.122 for patients initiating EFV, and between 0.054 and 0.127 for patients initiating DRV/r.

**Fig 2:**
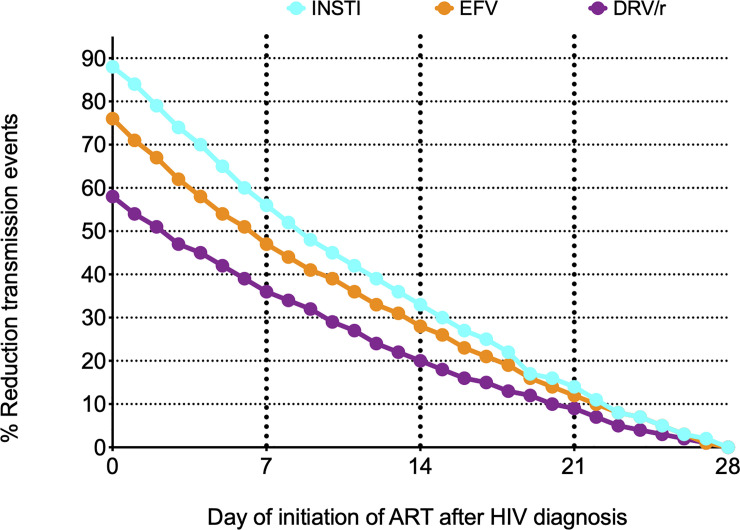
Relative reductions in simulated HIV-1 transmission events by day of initiation of ART and regimen used, using the initiation of ART on day 28 with a DRV/r-containing regimen as the referent case.

The relative reductions in simulated HIV-1 transmission events by day of initiation of ART and regimen used are shown in Fig 2 and supplementary material (S2 Table).

Initiation of ART on day 28 with a DRV/r-containing regimen was used as the referent case. In brief, in comparison with initiation of ART at day 28 with a DRV/r-containing regimen, the reduction in simulated HIV-transmission events with initiation of ART on day 0 with INSTI, EFV, or DRV/r was 88%, 76%, and 58%, respectively. Regarding the absolute number of simulated transmission events, initiating ART with EFV on day 0 was similar to starting ART with an INSTI on day 3, and starting ART with DRV/r on day 0 was similar to starting ART with an INSTI on day 7.

### Sensitivity analyses

The results of the three sensitivity analyses − sexual activity, simulated HIV-1 transmission events, and reduction of HIV-transmission events using initiation of ART at day 28 with DRV/r as the referent case − did not change the results found in the base case scenario; see supplementary material (S3, S4, S5, S6 and S7 Tables).

## Discussion

In a previous mathematical model, we found that INSTI-based regimens provide advantages over both EFV- and DRVr-based initial ART in the reduction of HIV-1 transmission risk from anal intercourse in HIV-infected MSM [10]. In this new analysis, we interrogated the effect of progressively early and immediate initiation of ART after diagnosis on the probability of HIV-1 transmission events in treatment naïve HIV-1-infected MSM using the same mathematical model. We found that the number of simulated transmission events per patient in the first 8 weeks after ART initiation increased linearly between same-day ART and deferred initiation until day 28 in all three treatment arms with advantages for INSTI-based therapy over EFV and DRV/r. We also found that in comparison to the initiation of ART at day 28 with DRV/r, the reduction in simulated HIV-transmission events with same-day ART with INSTI, EFV, or DRV/r was 88%, 76%, and 58%, respectively.

The finding that initiating ART with INSTI-based regimens can potentially reduce HIV transmission risk significantly when compared to non-INSTI regimens has been corroborated by another modeling study from British Columbia [17]; but to the best of our knowledge, this is the first mathematical model showing that ART initiation rapidly after diagnosis of HIV-1 infection in MSM has the potential to impact horizontal transmission of the infection. These findings support the ongoing study of rapid-start programs, particularly evaluating longer-term outcomes of maintenance of virologic suppression and interventions to support scale-up of such strategies; the findings also further support the in-progress global roll-out of INSTI-based therapy as first line, replacing EFV-based regimens. Besides the rapid initiation of ART, providing the necessary support (insurance, counseling, etc.) is key for long-term retention in care of the patients.

Our model is limited because HIV negative partners were modelled from the data from the START trial, without considering the HIV prevalence in the hypothetical cohort population. Besides, HIV-1 viral load decay kinetics were derived from pivotal clinical trials in which participants are often highly motivated and represent a population more likely to benefit from an intervention than would be seen in clinical practice. This, however, serves to contextualize the findings as an upper bound of an effect that might be seen in real-world implementation settings. Importantly, we did not consider PrEP use among seronegative partners, which, if used widely and appropriately, could dramatically attenuate HIV transmissions overall. The model has significant strengths as well, including inputs from metanalyses and well-curated clinical trial data and using it to model populations of sexually active MSM whose sexual behaviors are also modelled on data from a large and geographically diverse randomized clinical trial of first ART initiation. Also, results of sensitivity analyses demonstrate the robustness of the model over a wide range of values in the model inputs and assumptions.

## Conclusions

The results of this mathematical simulation support the notion that immediate or rapid ART initiation after diagnosis of HIV-1 for MSM has the potential to impact horizontal transmission. Small, clinically meaningful statistically significant advantages of INSTI over EFV and DRVr were found. Rapid, if not same-day initiation of INSTI-based ART to newly diagnosed HIV-infected individuals, has the potential for substantial public health benefits from decreases in secondary transmission events.

## Supporting information

S1 Fig Model structure. (DOCX)

S2 Fig Study design. (DOCX)

S1 Table HIV-1 transmission events. Base case scenario.
(DOCX)

S2 Table Relative reduction in simulated HIV-1 transmission events according to day of initiation of ART taking initiation at day 28 with DRV/r as reference. Base case scenario.
(DOCX)

S3 Table Simulated sexual activity and HIV-1 transmission events after initiation of ART, for the full week 0 to 8 period, in the three treatment arms (INSTI, EFV, and DRV/r) parametrized according to the sexual risk behavior questionnaire in MSM recruited in the START trial. Sensitivity analyses 1, 2, and 3.
(DOCX)

S4 Table HIV-1 transmission events. Sensitivity analysis 1.
(DOCX)

S5 Table HIV-1 transmission events. Sensitivity analysis 2.
(DOCX)

S6 Table HIV-1 transmission events. Sensitivity analysis 3.
(DOCX)

S7 Table Relative reduction in simulated HIV-1 transmission events according to day of initiation of ART taking initiation at day 28 with DRV/r as reference. Sensitivity analyses 1, 2, and 3.
(DOCX)
