# Supplementary figures and images for: Quantifying the reduction in sexual transmission of HIV-1 among MSM by early initiation of ART: A mathematical model
Source: PLoS One. 2020 Jul 20;15(7):e0236032. doi: 10.1371/journal.pone.0236032 (PMC7371210; doi:10.1371/journal.pone.0236032)

**S1 Fig.** Model structure


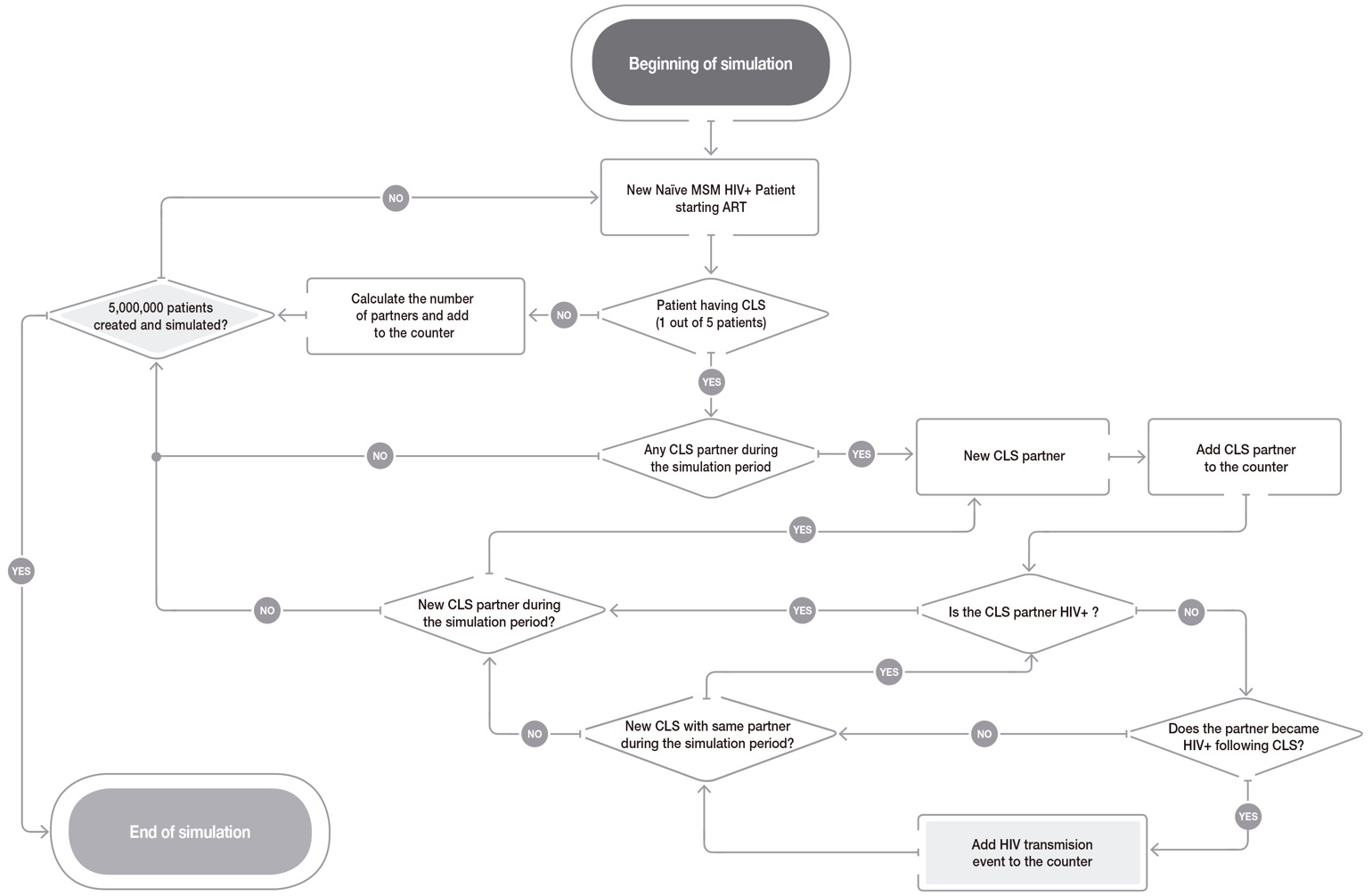

Supplement: S1 Fig — (DOCX) [file pone.0236032.s001.docx]

**S2 Fig**. Study design


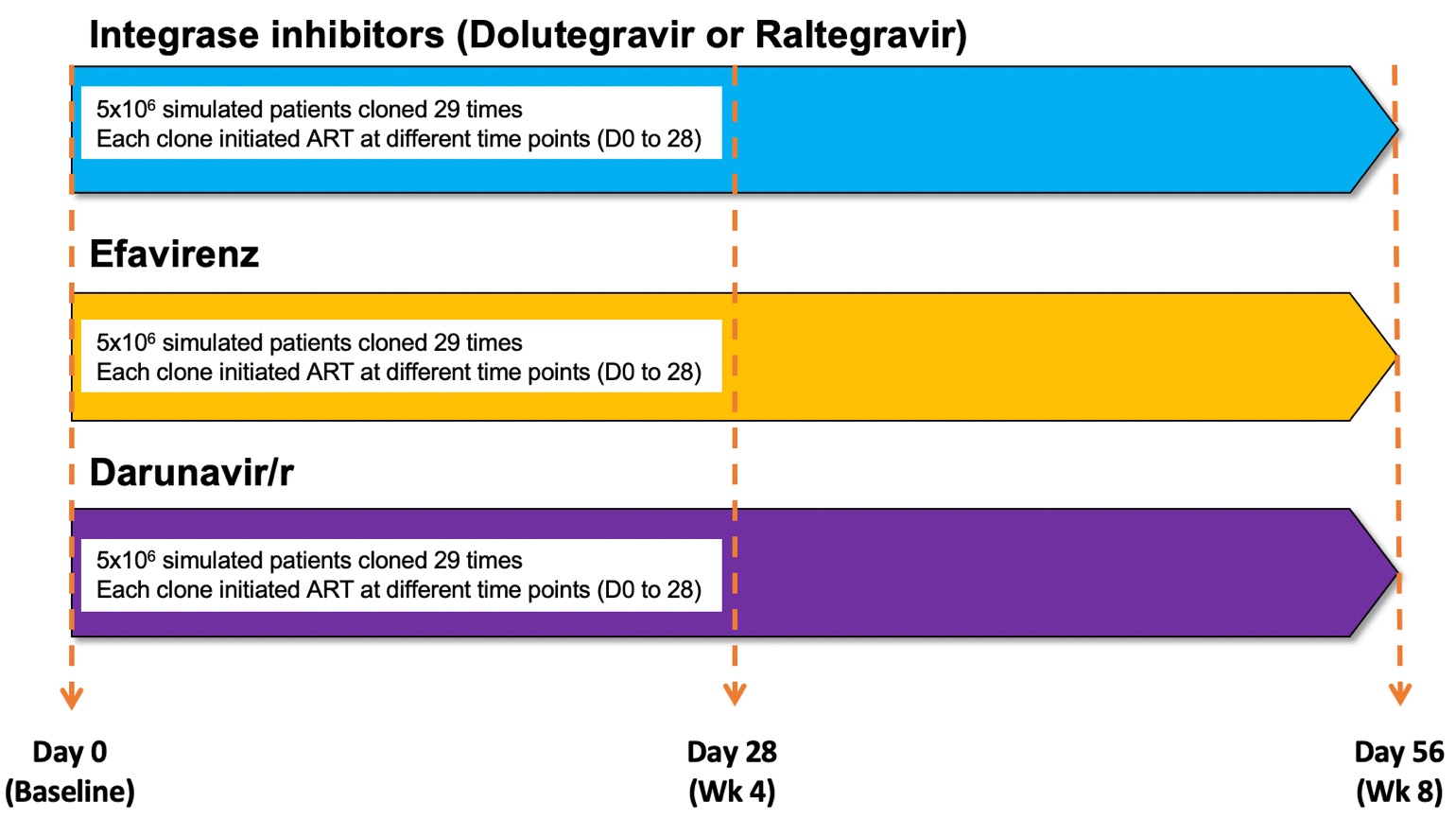

Supplement: S2 Fig — (DOCX) [file pone.0236032.s002.docx]
