# Supplementary material for: Quantifying the reduction in sexual transmission of HIV-1 among MSM by early initiation of ART: A mathematical model
Source: PLoS One. 2020 Jul 20;15(7):e0236032. doi: 10.1371/journal.pone.0236032 (PMC7371210; doi:10.1371/journal.pone.0236032)
Supplement: S2 Table — Base case scenario. (DOCX) [file pone.0236032.s004.docx]

**S2 Table.** Relative reduction in simulated HIV-1 transmission events according to day of initiation of ART taking initiation at day 28 with DRV/r as reference. Base case scenario.

| **Base case scenario** | | | |
| --- | --- | --- | --- |
| **Day of initiation of ART** | **% reduction in HIV-1 transmission events** | | |
|  | **INSTI** | **EFV** | **DRV/r** |
| 0 | 88 | 76 | 58 |
| 1 | 84 | 71 | 54 |
| 2 | 79 | 67 | 51 |
| 3 | 74 | 62 | 47 |
| 4 | 70 | 58 | 45 |
| 5 | 65 | 54 | 42 |
| 6 | 60 | 51 | 39 |
| 7 | 56 | 47 | 36 |
| 8 | 52 | 44 | 34 |
| 9 | 48 | 41 | 32 |
| 10 | 45 | 39 | 29 |
| 11 | 42 | 36 | 27 |
| 12 | 39 | 33 | 24 |
| 13 | 36 | 31 | 22 |
| 14 | 33 | 28 | 20 |
| 15 | 30 | 26 | 18 |
| 16 | 27 | 23 | 16 |
| 17 | 25 | 21 | 15 |
| 18 | 22 | 19 | 13 |
| 19 | 17 | 16 | 12 |
| 20 | 16 | 14 | 10 |
| 21 | 14 | 12 | 9 |
| 22 | 11 | 10 | 7 |
| 23 | 8 | 8 | 5 |
| 24 | 7 | 7 | 4 |
| 25 | 5 | 5 | 3 |
| 26 | 3 | 3 | 2 |
| 27 | 2 | 1 | 1 |
| 28 | 0 | 0 | 0 |
