# Supplementary material for: Quantifying the reduction in sexual transmission of HIV-1 among MSM by early initiation of ART: A mathematical model
Source: PLoS One. 2020 Jul 20;15(7):e0236032. doi: 10.1371/journal.pone.0236032 (PMC7371210; doi:10.1371/journal.pone.0236032)
Supplement: S3 Table — Sensitivity analyses 1, 2, and 3. (DOCX) [file pone.0236032.s005.docx]

**S3 Table.** Simulated sexual activity and HIV-1 transmission events after initiation of ART, for the full week 0 to 8 period, in the three treatment arms (INSTI, EFV, and DRV/r) parametrized according to the sexual risk behavior questionnaire in MSM recruited in the START trial*.

|  | **Base case scenario** | | | **Sensitivity analysis 1** | | | **Sensitivity analysis 2** | | | **Sensitivity analysis 3** | | |
| --- | --- | --- | --- | --- | --- | --- | --- | --- | --- | --- | --- | --- |
| **Simulated sexual activity** | **INSTI** | **EFV** | **DRV/r** | **INSTI** | **EFV** | **DRV/r** | **INSTI** | **EFV** | **DRV/r** | **INSTI** | **EFV** | **DRV/r** |
| Patients who initiated ART | 5,000,000 | 5,000,000 | 5,000,000 | 5,000,000 | 5,000,000 | 5,000,000 | 5,000,000 | 5,000,000 | 5,000,000 | 5,000,000 | 5,000,000 | 5,000,000 |
| Patients who engaged in CLSD (20%) | 1,000,000 | 1,000,000 | 1,000,000 | 1,000,000 | 1,000,000 | 1,000,000 | 1,000,000 | 1,000,000 | 1,000,000 | 1,000,000 | 1,000,000 | 1,000,000 |
| Partners of patients who engaged in CLSD | 9,613,268 | 9,598,679 | 9,621,582 | 9,650,035 | 9,619,141 | 9,622,978 | 9,581,413 | 9,602,331 | 9,651,335 | 9,600,073 | 9,597,743 | 9,599,698 |
| Sexual encounters in patients who engaged in CLS-D | 29,599,902 | 29,642,836 | 29,615,359 | 29,610,838 | 29,599,497 | 29,741,982 | 29,650,777 | 29,626,436 | 29,463,500 | 30,346,270 | 30,272,885 | 30,393,442 |
| Partners per patient who engaged in CLSD | 1.92 | 1.92 | 1.92 | 1.93 | 1,92 | 1.92 | 1.92 | 1.92 | 1.93 | 1.92 | 1.92 | 1.92 |
| Sexual encounters per partner in patients who engaged in CLSD | 3.08 | 3.09 | 3.08 | 3.07 | 3.08 | 3.09 | 3.09 | 3.09 | 3.05 | 3.16 | 3.15 | 3.17 |

^*^MSM population is based in sexual activity report on START trial (only 20% of the MSM population have condomless sex with an HIV-1-discordant status partner). In addition, a very small number of intercourse events among MSM in the START trial were reported to be with women.

**Base case scenario**: probability of transmission according to the mean value of the β_0_ parameter in the Wilson equation.

**Sensitivity analysis 1**: probability of transmission according to the lower 95% confidence interval value of the β_0_ parameter in the Wilson equation.

**Sensitivity analysis 2**: probability of transmission according to the upper 95% confidence interval value of the β_0_ parameter in the Wilson equation.

**Sensitivity analysis 3**: probability of transmission considering that 10% of patients never presented for care after the first visit assuming that during the follow-up they did not take the medication

Abbreviations: ART, antiretroviral therapy; INSTI, integrase strand transfer inhibitor; EFV, efavirenz; DRV/r, darunavir/ritonavir; MSM, men who have sex with men; CLSD, condomless sex with an HIV-1-discordant status partner.
