# Supplementary material for: Quantifying the reduction in sexual transmission of HIV-1 among MSM by early initiation of ART: A mathematical model
Source: PLoS One. 2020 Jul 20;15(7):e0236032. doi: 10.1371/journal.pone.0236032 (PMC7371210; doi:10.1371/journal.pone.0236032)
Supplement: S5 Table — Sensitivity analysis 2. (DOCX) [file pone.0236032.s007.docx]

**S5 Table.** HIV-1 Transmission Events. Sensitivity analysis 2*

| **INTEGRASE STRAND-TRANSFER INHIBITORS** | | |  | **EFAVIRENZ** | | |  | **DARUNAVIR/R** | | |
| --- | --- | --- | --- | --- | --- | --- | --- | --- | --- | --- |
| **Treatment**  **Delay (days)** | **Transmitted infections** | |  | **Treatment**  **Delay (days)** | **Transmitted infections** | |  | **Treatment**  **Delay (days)** | **Transmitted infections** | |
|  | **Per 5x10^6^ simulated patients** | **Per patient** |  |  | **Per 5x10^6^ simulated patients** | **Per patient** |  |  | **Per 5x10^6^ simulated patients** | **Per patient** |
| None | 57706 | 0.023 |  | None | 147665 | 0.054 |  | None | 269531 | 0.082 |
| 1 | 79775 | 0.030 |  | 1 | 174808 | 0.060 |  | 1 | 291075 | 0.086 |
| 2 | 104878 | 0.036 |  | 2 | 202115 | 0.066 |  | 2 | 314032 | 0.090 |
| 3 | 127845 | 0.043 |  | 3 | 230117 | 0.071 |  | 3 | 334778 | 0.093 |
| 4 | 149426 | 0.048 |  | 4 | 255250 | 0.076 |  | 4 | 352437 | 0.097 |
| 5 | 171706 | 0.054 |  | 5 | 278773 | 0.080 |  | 5 | 371994 | 0.100 |
| 6 | 195214 | 0.060 |  | 6 | 299177 | 0.085 |  | 6 | 387636 | 0.104 |
| 7 | 215748 | 0.065 |  | 7 | 319526 | 0.089 |  | 7 | 404692 | 0.107 |
| 8 | 235180 | 0.069 |  | 8 | 337693 | 0.092 |  | 8 | 419823 | 0.110 |
| 9 | 253149 | 0.073 |  | 9 | 356311 | 0.096 |  | 9 | 435446 | 0.113 |
| 10 | 269112 | 0.077 |  | 10 | 373307 | 0.100 |  | 10 | 451493 | 0.115 |
| 11 | 284396 | 0.080 |  | 11 | 391423 | 0.103 |  | 11 | 466917 | 0.118 |
| 12 | 300448 | 0.084 |  | 12 | 407062 | 0.107 |  | 12 | 482665 | 0.121 |
| 13 | 312869 | 0.088 |  | 13 | 421531 | 0.110 |  | 13 | 496647 | 0.123 |
| 14 | 329169 | 0.091 |  | 14 | 435441 | 0.113 |  | 14 | 509495 | 0.125 |
| 15 | 344417 | 0.094 |  | 15 | 451762 | 0.116 |  | 15 | 520553 | 0.127 |
| 16 | 357394 | 0.097 |  | 16 | 467410 | 0.119 |  | 16 | 532523 | 0.129 |
| 17 | 369594 | 0.101 |  | 17 | 481529 | 0.122 |  | 17 | 542835 | 0.131 |
| 18 | 382050 | 0.104 |  | 18 | 494844 | 0.124 |  | 18 | 551827 | 0.133 |
| 19 | 404588 | 0.108 |  | 19 | 509778 | 0.128 |  | 19 | 563455 | 0.135 |
| 20 | 411950 | 0.110 |  | 20 | 521473 | 0.129 |  | 20 | 570839 | 0.137 |
| 21 | 423557 | 0.113 |  | 21 | 534132 | 0.132 |  | 21 | 581116 | 0.138 |
| 22 | 434252 | 0.116 |  | 22 | 545484 | 0.134 |  | 22 | 592219 | 0.140 |
| 23 | 449265 | 0.118 |  | 23 | 558214 | 0.136 |  | 23 | 603557 | 0.141 |
| 24 | 454064 | 0.120 |  | 24 | 568180 | 0.138 |  | 24 | 609038 | 0.143 |
| 25 | 464047 | 0.122 |  | 25 | 578295 | 0.139 |  | 25 | 617414 | 0.144 |
| 26 | 473358 | 0.124 |  | 26 | 589100 | 0.141 |  | 26 | 623836 | 0.146 |
| 27 | 481894 | 0.126 |  | 27 | 598991 | 0.143 |  | 27 | 630528 | 0.147 |
| 28 | 490207 | 0.128 |  | 28 | 608037 | 0.144 |  | 28 | 637288 | 0.148 |

***Sensitivity analysis 2**: probability of transmission according to the upper 95% confidence interval value of the β_0_ parameter in the Wilson equation.
