# Supplementary material for: Quantifying the reduction in sexual transmission of HIV-1 among MSM by early initiation of ART: A mathematical model
Source: PLoS One. 2020 Jul 20;15(7):e0236032. doi: 10.1371/journal.pone.0236032 (PMC7371210; doi:10.1371/journal.pone.0236032)
Supplement: S6 Table — Sensitivity analysis 3. (DOCX) [file pone.0236032.s008.docx]

**S6 Table.** HIV-1 Transmission Events. Sensitivity analysis 3*

| **INTEGRASE STRAND-TRANSFER INHIBITORS** | | |  | **EFAVIRENZ** | | |  | **DARUNAVIR/R** | | |
| --- | --- | --- | --- | --- | --- | --- | --- | --- | --- | --- |
| **Treatment**  **Delay (days)** | **Transmitted infections** | |  | **Treatment**  **Delay (days)** | **Transmitted infections** | |  | **Treatment**  **Delay (days)** | **Transmitted infections** | |
|  | **Per 5x10^6^ simulated patients** | **Per patient** |  |  | **Per 5x10^6^ simulated patients** | **Per patient** |  |  | **Per 5x10^6^ simulated patients** | **Per patient** |
| None | 115355 | 0,02 |  | None | 200603 | 0,04 |  | None | 312414 | 0,06 |
| 1 | 136687 | 0,03 |  | 1 | 229502 | 0,05 |  | 1 | 335075 | 0,07 |
| 2 | 156664 | 0,03 |  | 2 | 257287 | 0,05 |  | 2 | 355140 | 0,07 |
| 3 | 176331 | 0,04 |  | 3 | 281676 | 0,06 |  | 3 | 376132 | 0,08 |
| 4 | 197001 | 0,04 |  | 4 | 304156 | 0,06 |  | 4 | 393782 | 0,08 |
| 5 | 217769 | 0,04 |  | 5 | 325490 | 0,07 |  | 5 | 410229 | 0,08 |
| 6 | 237187 | 0,05 |  | 6 | 343969 | 0,07 |  | 6 | 427554 | 0,09 |
| 7 | 254797 | 0,05 |  | 7 | 362625 | 0,07 |  | 7 | 442859 | 0,09 |
| 8 | 268663 | 0,05 |  | 8 | 380126 | 0,08 |  | 8 | 457460 | 0,09 |
| 9 | 283424 | 0,06 |  | 9 | 395989 | 0,08 |  | 9 | 470870 | 0,09 |
| 10 | 298429 | 0,06 |  | 10 | 413035 | 0,08 |  | 10 | 484688 | 0,10 |
| 11 | 315037 | 0,06 |  | 11 | 430316 | 0,09 |  | 11 | 498381 | 0,10 |
| 12 | 332840 | 0,07 |  | 12 | 446849 | 0,09 |  | 12 | 512359 | 0,10 |
| 13 | 349162 | 0,07 |  | 13 | 464263 | 0,09 |  | 13 | 524673 | 0,10 |
| 14 | 364680 | 0,07 |  | 14 | 480603 | 0,10 |  | 14 | 536425 | 0,11 |
| 15 | 378412 | 0,08 |  | 15 | 494384 | 0,10 |  | 15 | 548003 | 0,11 |
| 16 | 389958 | 0,08 |  | 16 | 505660 | 0,10 |  | 16 | 561284 | 0,11 |
| 17 | 402034 | 0,08 |  | 17 | 518745 | 0,10 |  | 17 | 572899 | 0,11 |
| 18 | 413569 | 0,08 |  | 18 | 531635 | 0,11 |  | 18 | 584029 | 0,12 |
| 19 | 430162 | 0,09 |  | 19 | 546613 | 0,11 |  | 19 | 595094 | 0,12 |
| 20 | 435926 | 0,09 |  | 20 | 557571 | 0,11 |  | 20 | 605301 | 0,12 |
| 21 | 447550 | 0,09 |  | 21 | 571007 | 0,11 |  | 21 | 614132 | 0,12 |
| 22 | 456984 | 0,09 |  | 22 | 582525 | 0,12 |  | 22 | 622320 | 0,12 |
| 23 | 472284 | 0,09 |  | 23 | 595587 | 0,12 |  | 23 | 630870 | 0,13 |
| 24 | 477405 | 0,10 |  | 24 | 603634 | 0,12 |  | 24 | 636705 | 0,13 |
| 25 | 487788 | 0,10 |  | 25 | 613519 | 0,12 |  | 25 | 643219 | 0,13 |
| 26 | 497742 | 0,10 |  | 26 | 623868 | 0,12 |  | 26 | 648904 | 0,13 |
| 27 | 505725 | 0,10 |  | 27 | 632585 | 0,13 |  | 27 | 656033 | 0,13 |
| 28 | 512211 | 0,10 |  | 28 | 640617 | 0,13 |  | 28 | 663638 | 0,13 |

***Sensitivity analysis 3**: probability of transmission considering that 10% of patients never presented for care after the first visit assuming that during the follow-up they did not take the medication.
