# Supplementary material for: Quantifying the reduction in sexual transmission of HIV-1 among MSM by early initiation of ART: A mathematical model
Source: PLoS One. 2020 Jul 20;15(7):e0236032. doi: 10.1371/journal.pone.0236032 (PMC7371210; doi:10.1371/journal.pone.0236032)
Supplement: S7 Table — Sensitivity analyses 1, 2, and 3. (DOCX) [file pone.0236032.s009.docx]

**S7 Table.** Relative reduction in simulated HIV-1 transmission events according to day of initiation of ART taking initiation at day 28 with DRV/r as reference

| **Base case scenario** | | | | **Sensitivity analysis 1** | | | | **Sensitivity analysis 2** | | | | **Sensitivity analysis 3** | | | |
| --- | --- | --- | --- | --- | --- | --- | --- | --- | --- | --- | --- | --- | --- | --- | --- |
| **Day of initiation**  **of ART** | **% reduction in HIV-1**  **transmission events** | | | **Day of initiation**  **of ART** | **% reduction in HIV-1**  **transmission events** | | | **Day of initiation**  **of ART** | **% reduction in HIV-1**  **transmission events** | | | **Day of initiation**  **of ART** | **% reduction in HIV-1**  **transmission events** | | |
|  | **INSTI** | **EFV** | **DRV/r** |  | **INSTI** | **EFV** | **DRV/r** |  | **INSTI** | **EFV** | **DRV/r** |  | **INSTI** | **EFV** | **DRV/r** |
| 0 | 88 | 76 | 58 | 0 | 92 | 86 | 74 | 0 | 82 | 62 | 45 | 0 | 77 | 69 | 53 |
| 1 | 84 | 71 | 54 | 1 | 88 | 82 | 69 | 1 | 77 | 58 | 42 | 1 | 73 | 64 | 50 |
| 2 | 79 | 67 | 51 | 2 | 83 | 77 | 65 | 2 | 71 | 55 | 39 | 2 | 69 | 60 | 46 |
| 3 | 74 | 62 | 47 | 3 | 78 | 72 | 61 | 3 | 66 | 51 | 37 | 3 | 66 | 56 | 43 |
| 4 | 70 | 58 | 45 | 4 | 73 | 68 | 57 | 4 | 62 | 47 | 34 | 4 | 62 | 53 | 41 |
| 5 | 65 | 54 | 42 | 5 | 70 | 64 | 53 | 5 | 57 | 44 | 32 | 5 | 57 | 49 | 38 |
| 6 | 60 | 51 | 39 | 6 | 66 | 59 | 49 | 6 | 53 | 41 | 30 | 6 | 54 | 46 | 36 |
| 7 | 56 | 47 | 36 | 7 | 62 | 55 | 46 | 7 | 49 | 39 | 28 | 7 | 50 | 43 | 33 |
| 8 | 52 | 44 | 34 | 8 | 57 | 51 | 43 | 8 | 46 | 36 | 26 | 8 | 48 | 41 | 31 |
| 9 | 48 | 41 | 32 | 9 | 54 | 48 | 41 | 9 | 43 | 33 | 24 | 9 | 45 | 38 | 29 |
| 10 | 45 | 39 | 29 | 10 | 50 | 45 | 38 | 10 | 40 | 31 | 22 | 10 | 42 | 36 | 27 |
| 11 | 42 | 36 | 27 | 11 | 46 | 41 | 35 | 11 | 37 | 29 | 20 | 11 | 38 | 33 | 25 |
| 12 | 39 | 33 | 24 | 12 | 43 | 38 | 32 | 12 | 34 | 26 | 18 | 12 | 35 | 30 | 23 |
| 13 | 36 | 31 | 22 | 13 | 40 | 35 | 30 | 13 | 31 | 24 | 17 | 13 | 32 | 28 | 21 |
| 14 | 33 | 28 | 20 | 14 | 37 | 32 | 28 | 14 | 29 | 21 | 15 | 14 | 29 | 25 | 19 |
| 15 | 30 | 26 | 18 | 15 | 34 | 29 | 25 | 15 | 26 | 19 | 14 | 15 | 26 | 23 | 17 |
| 16 | 27 | 23 | 16 | 16 | 31 | 27 | 23 | 16 | 24 | 18 | 12 | 16 | 24 | 21 | 15 |
| 17 | 25 | 21 | 15 | 17 | 28 | 24 | 20 | 17 | 21 | 16 | 11 | 17 | 22 | 19 | 14 |
| 18 | 22 | 19 | 13 | 18 | 25 | 21 | 18 | 18 | 19 | 14 | 10 | 18 | 19 | 17 | 12 |
| 19 | 17 | 16 | 12 | 19 | 21 | 18 | 16 | 19 | 16 | 11 | 9 | 19 | 16 | 15 | 10 |
| 20 | 16 | 14 | 10 | 20 | 20 | 16 | 14 | 20 | 14 | 10 | 8 | 20 | 15 | 13 | 9 |
| 21 | 14 | 12 | 9 | 21 | 17 | 14 | 12 | 21 | 12 | 9 | 6 | 21 | 13 | 11 | 7 |
| 22 | 11 | 10 | 7 | 22 | 14 | 12 | 10 | 22 | 10 | 7 | 5 | 22 | 11 | 9 | 6 |
| 23 | 8 | 8 | 5 | 23 | 9 | 10 | 8 | 23 | 7 | 6 | 4 | 23 | 8 | 7 | 5 |
| 24 | 7 | 7 | 4 | 24 | 9 | 8 | 7 | 24 | 6 | 5 | 3 | 24 | 7 | 6 | 4 |
| 25 | 5 | 5 | 3 | 25 | 6 | 6 | 5 | 25 | 5 | 3 | 2 | 25 | 5 | 4 | 3 |
| 26 | 3 | 3 | 2 | 26 | 4 | 4 | 4 | 26 | 3 | 2 | 1 | 26 | 3 | 3 | 2 |
| 27 | 2 | 1 | 1 | 27 | 2 | 2 | 2 | 27 | 1 | 1 | 1 | 27 | 1 | 1 | 1 |
| 28 | 0 | 0 | 0 | 28 | 0 | 0 | 0 | 28 | 0 | 0 | 0 | 28 | 0 | 0 | 0 |

**Base case scenario**: probability of transmission according to the mean value of the β_0_ parameter in the Wilson equation.

**Sensitivity analysis 1**: probability of transmission according to the lower 95% confidence interval value of the β_0_ parameter in the Wilson equation.

**Sensitivity analysis 2**: probability of transmission according to the upper 95% confidence interval value of the β_0_ parameter in the Wilson equation.

**Sensitivity analysis 3**: probability of transmission considering that 10% of patients never presented for care after the first visit assuming that during the follow-up they did not take the medication
